# Competition and Facilitation between a Disease and a Predator in a Stunted Prey Population

**DOI:** 10.1371/journal.pone.0132251

**Published:** 2015-07-06

**Authors:** Maarten C. Boerlijst, André M. de Roos

**Affiliations:** Theoretical Ecology, Institute for Biodiversity and Ecosystem Dynamics, University of Amsterdam, P.O. Box 94248, 1090 GE, Amsterdam, The Netherlands; Shanxi University, CHINA

## Abstract

The role of diseases and parasites has received relatively little attention in modelling ecological dynamics despite mounting evidence of their importance in structuring communities. In contrast to predators, parasites do not necessarily kill their host but instead they may change host life history. Here, we study the impact of a parasite that selectively infects juvenile prey individuals and prevents them from maturing into adults. The model is inspired by the *Ligula intestinalis* tape worm and its cyprinid fish host *Rutilis rutilis*. We demonstrate that the parasite can promote as well as demote the so-called stunting in its host population, that is, the accumulation of juvenile prey, which leads to strong exploitation competition and consequently to a bottleneck in maturation. If competition between infected and uninfected individuals is strong, stunting will be enhanced and bistability between a stunted and non-stunted prey population occurs. In this case, the disease competes with the predator of its host species, possibly leading to predator extinction. In contrast, if the competition between infected and uninfected individuals is weak, the stunting is relieved, and epi-zoonotic cycles will occur, with recurrent epidemic outbreaks. Here, the disease facilitates the predator, and predator density will be substantially increased. We discuss the implications of our results for the dynamics and structure of the natural *Ligula-Roach* system.

## Introduction

Diseases and parasites play a major role in most ecosystem dynamics; yet, they have received relatively little attention in ecological modelling [1–3]. Traditionally, ecologists have mostly considered disease outbreaks as disturbances rather than as an integral part of the ecosystem dynamics [4]. However, there is mounting evidence that diseases and parasites play a vital role in the stability and dynamics of most ecosystems [5–9].

In this paper we will consider the effects of a disease that specifically infects juvenile individuals and subsequently prevents the infected individual from producing offspring. The work is inspired by the interaction between the tape worm *Ligula intestinalis* and its cyprinid fish host species roach (*Rutilis rutilis*). The parasite has a complex life cycle, involving three host species. The final host species of the parasite are fish eating birds, such as cormorant (*Phalacrocorax carbo*), grey herron (*Ardea cinerea*), and great crested grebe (*Podiceps cristatus*) [10]. In this final host the parasite completes its sexual cycle and produces eggs, which are shed to the water via the faeces of the birds. The larvae of the parasite are eaten by copepods, which in turn are a primary food source for the juvenile fish. After a fish is infected, the parasite settles and grows in the host gut system and may obtain a staggering weight of up to half the host weight [11]. Surprisingly, the physical condition of such a fish-parasite combination can be not noticeably different from uninfected individuals [12]. However, the consequences for the infected fish are severe, as the parasites supress gonad development and consequently prevent the individual from maturing. Even infection with a single parasite can act to sterilize the infected fish [12]. Furthermore, the parasite might induce behavioural changes, such as directing the infected fish to move to the littoral zone, where it can be caught more easily by the fish eating birds and thus complete the parasite life cycle [13,14].

Also the consequences of the parasite infection on the host population dynamics can be extensive. A well-documented case are the recurrent *Ligula* parasite outbreaks in the roach population of lake Slapton Ley [11,15,16]. The roach population in this lake shows a tendency to build-up a so-called stunted population. In this population state, small juvenile individuals are very numerous. This causes exploitation competition amongst juveniles to be so fierce, that individual growth rate substantially slows down, effectively preventing juveniles from reaching the size necessary for maturation [16,17].

In this stunted population situation, the *Ligula* parasite can cause a large epidemic, reaching a prevalence of more than 70% of all juveniles being infected [11]. This in turn seems to act to relieve the stuntedness in the juvenile population, causing a reduction in the juvenile population size, with a subsequent acceleration in juvenile growth and maturation, accompanied by a strong reduction of the parasite prevalence. However, hereafter, the stuntedness in the host population slowly rebuilds, after which a second epidemic of the parasite can occur. In the case of the Slapton Ley dataset, such recurrent epidemics were recorded three times, spanning a total period of 31 years [11].

In this paper we will study a mathematical model to investigate the interplay between ecological population dynamics of a potentially stunted population and a disease. Furthermore, we will study the interaction between the disease and a natural predator of the host. In the *Ligula*–Roach system there are several predators on Roach, including Northern Pike (*Esox Lucius*) and European Perch (*Perca fluvitilis)*. Often, such predator species are of commercial or recreational interest for fisherman, and stunting of the prey species can greatly affect predator numbers or predator quality. Here, we will study the potential indirect influence of a prey disease on the predator species. For the ecological baseline, we will use a model that we have introduced before for the dynamics of a structured prey population and a predator that attacks adult prey [18,19]. In this model a stunted juvenile prey population can build up due to a maturation bottleneck, and it has a parameter region of bistability between a stunted and non-stunted equilibrium state [19]. To this system we will add a disease that specifically infects juvenile individuals, and that acts to sterilize these individuals and prevents maturation. In this way we can study the indirect interaction between a predator and a disease, that both attack the same host species, but each targeting a different host life stage. For mathematical simplicity, we model a disease that spreads through direct contact. This differs from the parasite infection in the *Ligula*-Roach system, where the parasite has to pass through two additional host stages. Our simplified model would roughly correspond to a constancy assumption in the copepod and bird populations. Furthermore, we do not explicitly account for individual parasite load, but instead we only distinguish between infected and uninfected individuals.

Our main objective in this paper is to study the effects of a disease that infects and sterilizes juvenile individuals on the population dynamics of its host species. In particular, we will focus on a system where the host population is in a stunted population state, which is strongly dominated by juvenile individuals and where individual growth rate and maturation are strongly reduced. We will map the dynamic consequences of introducing such a disease in a stunted host population state. It will turn out that these consequences strongly depend on the ability of infected juveniles to compete with uninfected conspecifics. We will discuss the biological implications of our results.

## Methods

In this paper, we use a previously described model system consisting of a stage-structured prey population, distinguishing between juvenile and adult individuals, and a predator that preys upon adult prey individuals [18,19]. To this system we add a disease that specifically targets juvenile individuals. The model consists of four ordinary differential equations for, respectively, susceptible juvenile (*J*_*S*_), infected juvenile (*J*_*I*_), adult (*A*), and predator density (*P*):

$$\frac{dJ_{S}}{dt}=bA-m(J)J_{S}-\beta J_{S}J_{I}-\mu_{J}J_{S}$$ (1)

$$\frac{dJ_{I}}{dt}=\beta J_{S}J_{I}-(\mu_{J}+\alpha )J_{I}$$ (2)

$$\frac{dA}{dt}=m(J)J_{S}-nAP-\mu_{A}A$$ (3)

$$\frac{dP}{dt}=cnAP-\mu_{P}P$$ (4)

Here, parameters *μ*_*J*_, *μ*_*A*_ and *μ*_*P*_ are death rates, *b* is adult reproduction rate, *β* is infectivity, α is virulence (i.e. additional mortality due to disease), *n* is the predator attack rate, and *c* is a conversion factor. The function *m(J)* denotes the maturation rate, which is specified by:

$$m(J)=\varphi /[1+d{\left(J_{S}+\delta J_{I}\right)}^{2}]$$ (5)

At low juvenile density the maturation rate approximates the maximum rate *φ*. Due to competition amongst juveniles maturation sharply decreases at higher juvenile density, reflecting a maturation bottleneck [17]. Parameter *d* denotes the strength of the exploitation competition between juvenile consumers. Parameter *δ* scales the relative contribution of infected juveniles to the juvenile competition. Note that in Eq (3) we use the assumption that only uninfected juveniles can mature into adults, as the disease sterilizes infected individuals and prevents them from maturing. Furthermore, infected juveniles cannot be cured from the parasite, so they can only disappear from the system through mortality. For numerical integration and bifurcation analysis we used the MatCont program [20]. This software can numerically compute and continue bifurcations. All reported dynamics regimes (i.c. equilibriums, limit cycles and bistable regions) where confirmed using numerical integration techniques.

## Results

### Bistability and stunted prey population in the absence of disease

**Fig 1:**
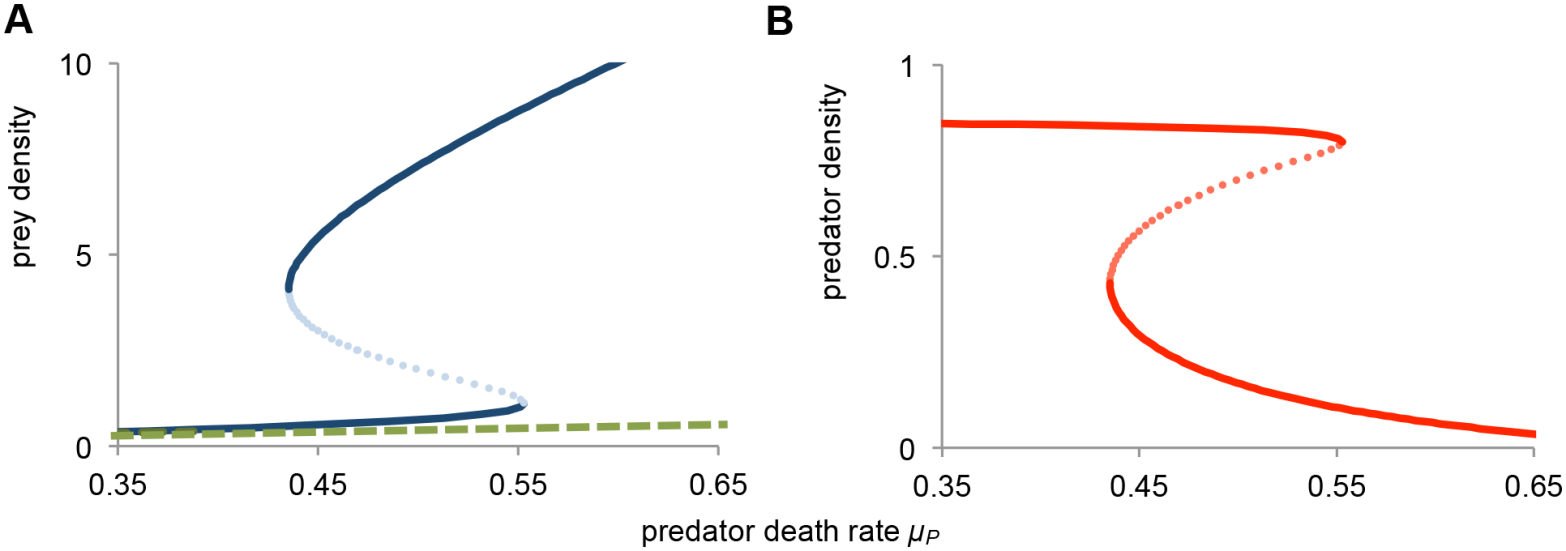
Bistability in the absence of disease. Bifurcation diagram as a function of predator death rate *μ*_*P*_. *(a)* Equilibrium prey density, juveniles *J*_*S*_ in solid line (blue), and adults *A* in dashed line (green), and (*b*) Equilibrium predator density *P* (red). The equilibrium curves exhibit a so-called catastrophe fold, with a bistable region for 0.435 < *μ*_*P*_ < 0.553. The unstable (saddle node) state is indicated with the dotted lines. Model parameters are *b* = 1, *c* = 1, *d* = 1, *n* = 1, φ = 1, μ_J_ = 0.05, *μ*_*A*_ = 0.1, and *μ*_*P*_ variable.

We first show the dynamics of our model in the absence of the disease. In Fig 1*a* and 1*b*, the equilibrium density of, respectively, prey and predator is plotted as a function of the predator death rate *μ*_*P*_. For intermediate predator death rate (i.c. 0.435 < *μ*_*P*_ < 0.553) there exists a region of bistability. Interestingly, in both alternative steady states the adult prey density is identical, as it is uniquely determined by the predator equilibrium; i.c. putting Eq (4) to zero gives $A=\frac{\mu_{P}}{cn}$ for both alternative steady states. In the equilibrium with high juvenile density, the corresponding predator density is small. Here, the prey population is stunted in the juvenile class and, consequently, maturation is strongly reduced. Consequently, the adult prey population in this case is mainly limited by the low maturation. In contrast, in the low juvenile equilibrium, the predator density is much larger. In this equilibrium, the total maturation into the adult population is increased, but this is balanced by the larger predation rate. In conclusion, in the absence of the disease the prey population can be caught in a stunted equilibrium, where high abundance of juvenile prey causes strong competition amongst juveniles, resulting in stunted growth and a maturation bottleneck. However, such a large juvenile prey population could serve as a target for a disease, which could potentially act to relieve the stuntedness of the prey population.

### Disease invasion threshold and endemic equilibrium

We now add the juvenile disease to the system. Depending on the disease parameters there exists a critical juvenile prey density above which the disease can invade the system. This critical density can be found by solving Eq (2) for $\frac{dJ_{I}}{dt}>0$, which gives:

$$J_{S}>\frac{\left(\mu_{J}+\alpha \right)}{\beta }$$ (6)

In the remainder of this paper we choose the disease parameters such that the disease can indeed invade the stunted population equilibrium. If there exists a steady state with the disease present, this so-called endemic equilibrium will be characterized by $J_{S}=\frac{\left(\mu_{J}+\alpha \right)}{\beta }$. The stability and region of attraction of this endemic equilibrium will depend on the specific properties of the disease, as defined by the parameters. In particular, it turns out that it is important to what extent sick juveniles are capable of competing with healthy juveniles. This property is modelled with parameter δ in the function for the maturation rate (Eq (5)), which scales the relative contribution of infected juveniles to the juvenile competition. We will first consider the two extreme cases, that is *δ* = 1, implying full competitive ability of sick individuals, and *δ* = 0, implying absence of competition between sick and healthy juveniles.

### Disease enhances bistability and population stuntedness, for the case of full competition between sick and healthy juveniles

**Fig 2:**
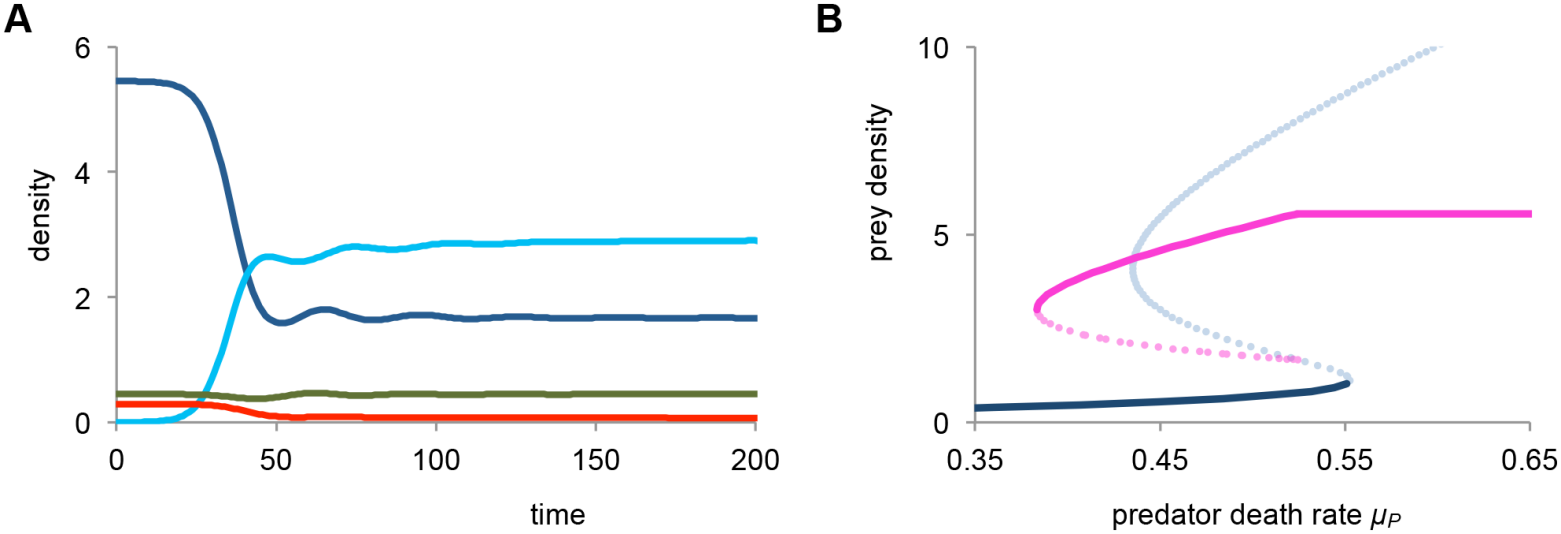
The disease can suppress the predator and enhance bistability and population stuntedness. *(a)* Dynamics after introduction of the disease in the stunted population state for *μ*_*P*._ = 0.45. Density of infected juveniles *J*_*I*_ are shown in light blue. Disease parameters are *β* = 0.06, *α* = 0.05, and δ = 1, indicating that sick individuals have full competitive ability. For other colors and parameters see Fig 1. Note the strong reduction in predator density after introduction of the disease. *(b)* Bifurcation diagram for total juvenile prey density (i.c. *J*_*S*_ *+J*_*I*_) as a function of predator death rate *μ*_*P*._ The solid blue line indicates the stable disease free state, and the solid purple line denotes the endemic state. Dashed lines indicate unstable equilibriums. Note that the introduction of the disease considerably increases the region of bistability.

We first consider the case of δ = 1, which implies that sick juveniles have full competitive ability against healthy juveniles. Because sick individuals compete with healthy individuals, but do not mature themselves, they can act to reinforce the stunted juvenile population bottleneck. In Fig 2*a* we show a typical example of the dynamics after introduction of the disease in the stunted population equilibrium. The disease quickly spreads through the juvenile population, and the system settles in an endemic equilibrium. The prevalence of the disease in this equilibrium is high at 64% of all juveniles being infected. Furthermore, the introduction of the disease strongly reduces the predator abundance, causing a roughly 4-fold reduction. This seems surprising, as the adult prey density does not change, and this is the food source for the predator. However, the adult prey density is now mainly controlled by the maturation bottleneck, which causes the predator equilibrium to be strongly reduced.

In Fig 2*b*, a bifurcation diagram is shown for the total juvenile prey density as a function of the predator death rate. The disease free stunted population equilibrium has now become unstable, because it can be invaded by the disease. Instead, there is a stable endemic equilibrium with the disease present at high prevalence. Notably, the stunted population equilibrium, and the accompanying bistability now persist for much lower predator death rates (up to *μ*_*P*_ = 0.383). Furthermore, the predator goes extinct for death rates exceeding *μ*_*P*_ = 0.524. Both results can be explained by the fact that there is a form of apparent competition between the disease and the predator. This causes the predator density to be reduced or even extinct after introduction of the disease, and the release of predator pressure acts to reinforce the stunted juvenile equilibrium.

### Disease causes relieve of stuntedness and recurrent epidemics, for the case of absence of competition between sick and healthy juveniles

**Fig 3:**
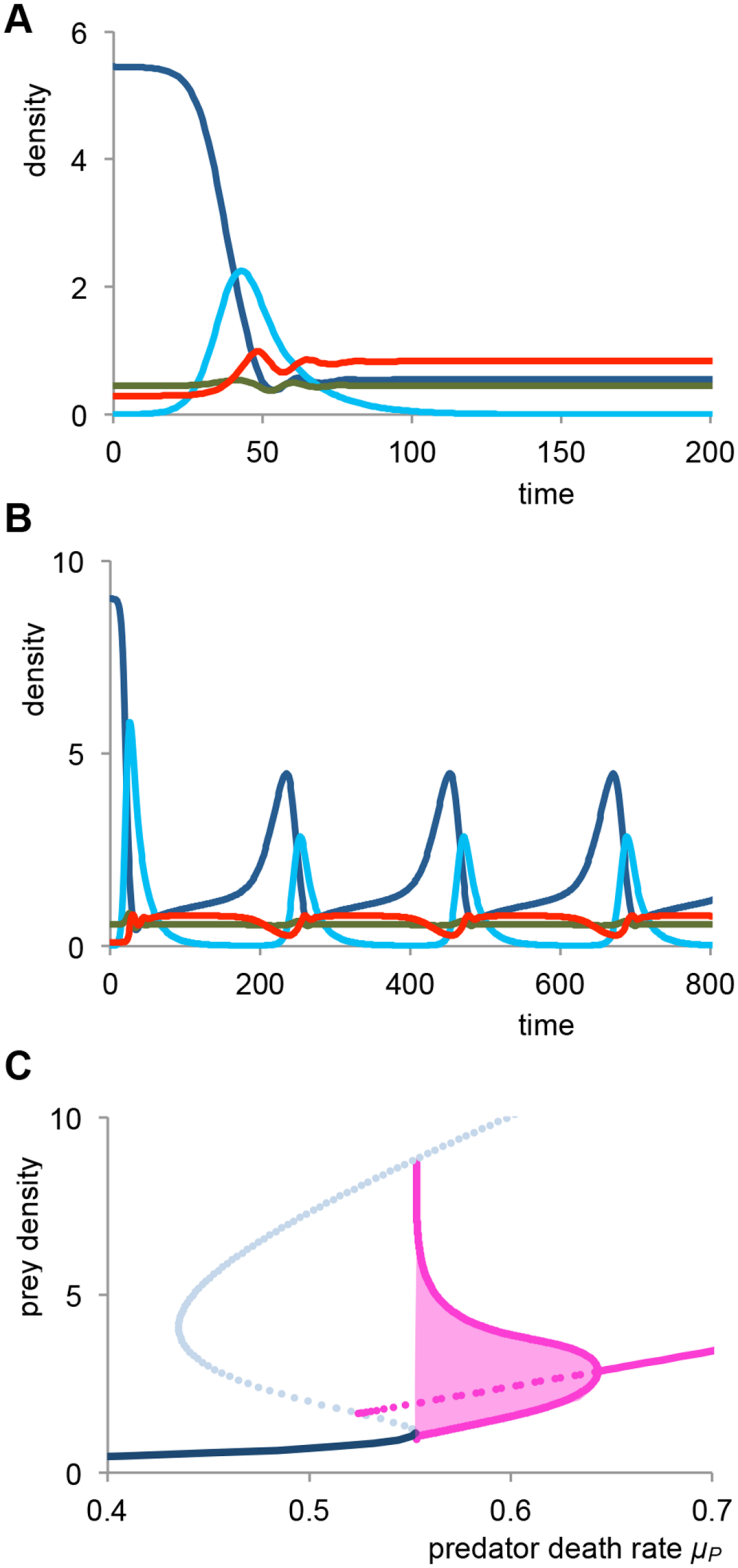
The disease can facilitate the predator, relieve stuntedness and induce recurrent epidemics. Parameters and colors are identical to Fig 2, except δ = 0, indicating that sick individuals do not effectively compete with healthy individuals. *(a)* Dynamics after introduction of the disease in the stunted population state for *μ*_*P*._ = 0.45. Here, the disease causes a single epidemic, after which the host population shifts to the non-stunted equilibrium and the disease goes extinct. *(b)* Dynamics after introduction of the disease in the stunted population state for *μ*_*P*._ = 0.56. After an initial large epidemic, the system settles in a limit cycle with recurrent epidemics of intermediate amplitude. Note that each epidemic is closely followed by an increase in predator density. *(c)* Bifurcation diagram for total juvenile prey density as a function of predator death rate *μ*_*P*._ The solid blue line indicates the stable disease free state, and the solid purple line denotes the endemic state. The red lines and area indicate the amplitude of the stable limit cycles. Dashed lines are unstable equilibriums.

Now we consider the case that infected juveniles do not effectively compete with healthy juveniles, i.c. *δ* = 0. This can e.g. be due to sick individuals being in a bad condition, or shifting their diet or habitat. In Fig 3*a*, invasion of the disease in the stunted population is shown for intermediate predator death rate of *μ*_*P*_ = 0.45. Here, the disease causes a single epidemic, but this induces the population to shift to the non-stunted equilibrium and consequently the disease goes extinct after the epidemic. In this case, the effect of the disease on the predator is positive, as the introduction of the disease causes a roughly threefold increase in the predator density. If we introduce the disease in the stunted population for an increased predator death rate of *μ*_*P*_ = 0.56, in Fig 3*b*, we obtain a new type of dynamics. In this case, after a large initial epidemic, the disease almost disappears, but for this predator death rate there does not exist a stable non-stunted population equilibrium. Consequently, after the epidemic, the population slowly rebuilds to the stunted equilibrium, but this in turn allows the disease to re-enter the population and cause a secondary epidemic. This process repeats itself, resulting in limit cycle behaviour with recurrent epidemics, with prevalence varying strongly between 1% and 75%. The introduction of the disease again has a strong positive effect on the predator, increasing predator density by a factor varying between 3-fold (at the start of the epidemic) and 8-fold (during and after the epidemic). However, the interaction between the disease and the predator in this case is complex, as the highest predator densities are actually reached in the periods where the disease is almost absent. Yet, also in this case the disease acts to facilitate the predator, as it prevents the prey population from getting stuck in the stunted equilibrium. Actually, the recovery of the predator population closely follows the epidemic outbreaks.

In Fig 3*c*, a bifurcation diagram is shown for the total juvenile prey density as a function of the predator death rate. For small predator death rate, *μ*_*P*_ < 0.553, the system has a single steady state, consisting of a non-stunted prey population that cannot be invaded by the disease. For intermediate predator death rates 0.553 ≤ *μ*_*P*_ < 0.643, the system exhibits stable recurrent epidemics (i.c. a limit cycle), where the amplitude of the epidemics decrease for increasing predator death rate. For large predator death rate, *μ*_*P*_ ≥ 0.643, after introduction of the disease the system reaches a stable endemic state, where the stuntedness of the prey population is largely reduced and, as a consequence, the predator is benefitting from the presence of the disease.

### Varying the disease invasion threshold

**Fig 4:**
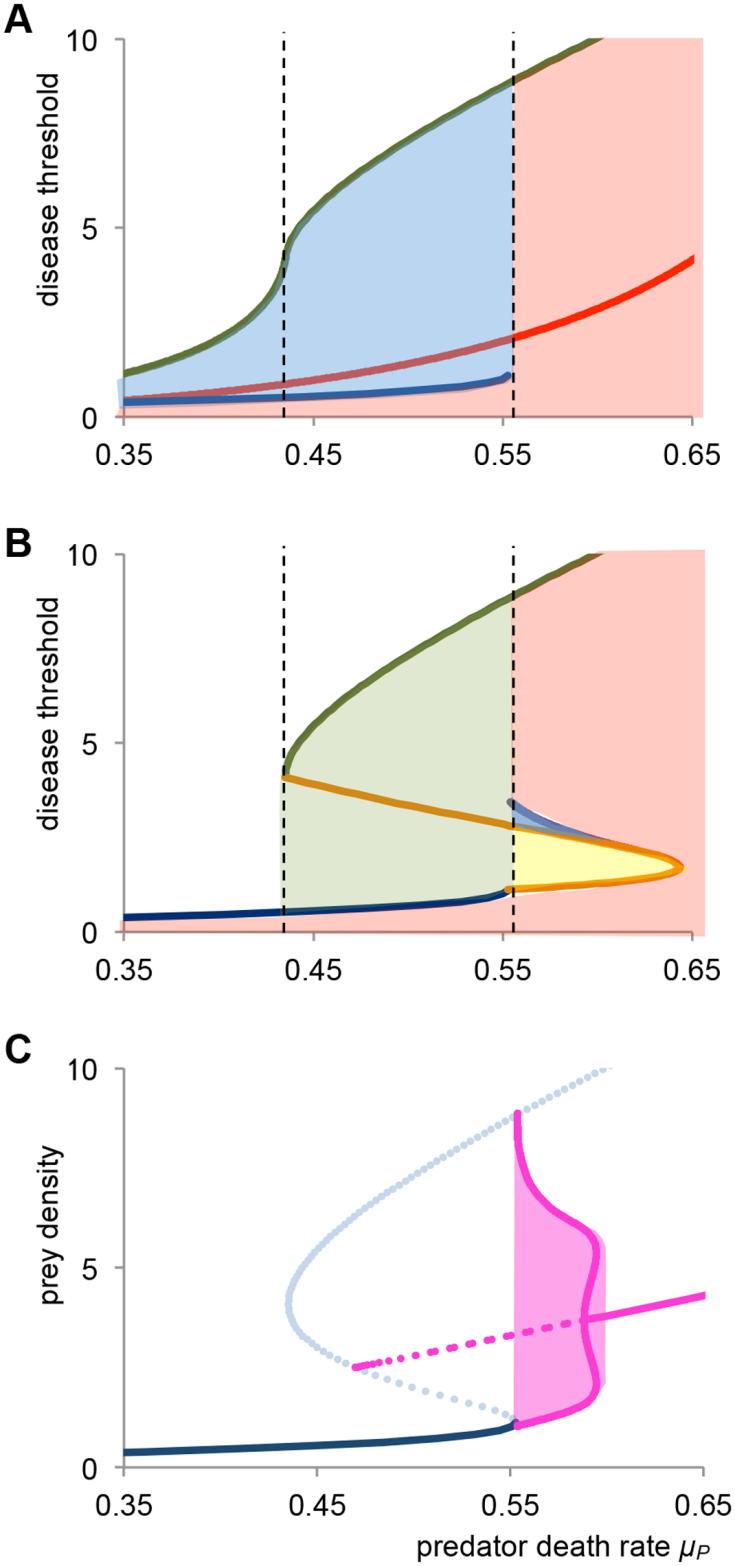
Dynamics for varying disease invasion threshold. Plotting dynamics as a function of predator death rate *μ*_*P*,_ and disease invasion threshold *(μ*_*J*_+*α)/β*. *(a)* Dynamics for the case of *δ* = 1. In the white area the disease cannot invade, and in the pink area there is a single endemic equilibrium. The blue area indicates bistability between a stunted endemic state and a non-stunted disease free state. The red line denotes predator extinction. The green and blue line are thresholds below which the disease can invade respectively, the stunted and non-stunted host population. The dashed black lines indicate the disease free region of bistability *(b)* Dynamics for the case of *δ* = 0. In the green area only the disease free non-stunted state is stable. The yellow area indicates stable recurrent epidemics, and in the purple area there is bistability of recurrent epidemics and an endemic state. The orange line is a Hopf bifurcation, either supercritical (solid line) or subcritical (dashed line). Other lines and areas are as described in Fig 4*a (c)* Bifurcation diagram for the subcritical Hopf bifurcation for *β* = 0.06. The bifurcation resembles that of Fig 3*c*, but now there is a region of bistability between an endemic state and a stable limit cycle for 0.588 < *μ*_*P*_ < 0.595. The red area denotes the amplitude of the stable limit cycle, and the dashed red line shows the minimum and maximum of the unstable limit cycle. For parameters see Fig 3.

Until now, we have fixed the invasion threshold for the disease at *J*_*S*_ = 1.67. Here, we explore what happens if we vary the disease invasion threshold. We demonstrate this by varying the disease infectivity *β*, but varying disease virulence *α*, will yield similar results. In Fig 4*a* we plot a two-parameter diagram for the case of δ = 1 (i.c. full competitive ability of infected juveniles), were we map the dynamics as a function of the predator death rate *μ*_*P*_ and the disease invasion threshold *(μ*_*J*_+*α)/β*. There exist three distinct areas that differ in dynamics. In the white area, the disease cannot invade, and the dynamics are identical to Fig 1. In the blue shaded area there is bistability between a stunted endemic state and a non-stunted disease free state. In the pink area, there is a single endemic equilibrium. The red line indicates the threshold below which the predator goes extinct in the endemic state. The green line indicates the threshold below which a stunted endemic state exists, and the blue line is the threshold below which a non-stunted endemic state exists. With increasing severity of the disease (i.c. increasing infectivity *β* and/or decreasing virulence *α*) the area of bistability increases, and the predator is reduced to lower densities or even extinction.

In Fig 4*b* a similar two-parameter diagram is plotted for the case of δ = 0 (i.c. infected individuals do not compete), where we again map the dynamics as a function of the predator death rate *μ*_*P*_ and the disease invasion threshold *(μ*_*J*_+*α)/β*. The white region is identical to the previous case and denotes failure of disease invasion. In the green region, the disease can invade the stunted population, but after a single epidemic the system shifts to the non-stunted state and the disease subsequently goes extinct. The pink region again is characterized by existence of a stable endemic equilibrium. In the yellow region, the system settles in a limit cycle with recurrent epidemics. This region is bounded by a Hopf bifurcation, from which the limit cycle originates, indicated by the solid orange line. Finally, in the blue area, there is bistability between an endemic state and recurrent epidemics. Here, the Hopf bifurcation is subcritical [21], meaning that from the Hopf an unstable limit cycle originates, which acts to separate the stable endemic state from the stable limit cycle. The unstable Hopf is indicated by the dashed orange line. In Fig 4*c* a bifurcation diagram is shown for this situation. The bifurcation is similar to that of Fig 3*c*, but now there is a small range between 0.588 ≤ *μ*_*P*_ < 0.595 where a stable endemic equilibrium coexists with a stable limit cycle with recurrent epidemics. The two attractors are separated by an unstable limit cycle that originates from the Hopf bifurcation. In conclusion, in the case that infected individuals do not compete (i.c. δ = 0) the disease has a positive effect on the predator density, and furthermore there is a considerable parameter region where the disease can relieve the stunted population into a stable limit cycle with recurrent epidemics.

## Discussion

In this paper we have demonstrated that a disease that specifically infects juvenile individuals and prevents their maturation can substantially affect the dynamics of the host population and its predator species. In particular, we investigated the effects of introducing such a disease in a stunted population, and we have shown that depending on disease parameters the disease can either increase or relieve population stuntedness. In particular, it turns out that a key disease property is whether infected individuals can still compete with their uninfected conspecifics. If infected juveniles have the same competitive ability as uninfected individuals, the disease acts to increase stuntedness, and introduction of the disease will act to decrease predator density or even cause predator extinction. If, on the other hand, infected individuals do not compete with uninfected conspecifics, the disease will act to relieve population stuntedness, and the predator will benefit from introduction of the disease. In this situation introduction of the disease can lead to recurrent epidemic outbreaks.

We studied a fairly simple structured prey population model that only distinguishes between juvenile and adult individuals. However, the model can easily be extended to include more prey stages or incorporate a continuous prey size distribution [18,22,23]. Interestingly, in these extended models the bistability and the stunted prey population can also be generated if the predator attacks small juveniles instead of adults. Our work was inspired by the *Ligula*-roach parasite-host interaction as documented in lake Slapton Ley [11]. In this system, recurrent epi-zoonotic cycles were observed with build-up of a stunted roach population that was subsequently released by a large outbreak of *Ligula* infection. Our model is capable of reproducing such recurrent epidemics, although the parameter region for this behaviour is restricted. This in fact would suggest that the competitive ability of infected individuals is small. We have checked that in our model the relative competitive ability of infected juveniles should be smaller than *δ* = 0.25, in order for recurrent epidemics to emerge. The relative good condition of infected fish seems to contradict this finding [12], but the behavioural changes in infected fish could cause that the direct competition with uninfected conspecifics is substantially reduced [13, 14].

As in most cases, the natural system has many more levels of complexity than are included in the current model. For instance, in lake Slapton Ley, the roach population is in competition with another fish species rudd [11]. In the absence of the parasite roach is dominant over rudd, but after the epidemic outbreaks the dominance is shifted and it takes a very long period for roach to regain control. This mechanism seems a possible candidate to explain the long timescale for the reoccurrence of the stunted roach population structure. Unfortunately, predator densities are not readily available for the Slapton Ley dataset. The model prediction would be that predator densities are largely enhanced just after the epidemic outbreaks, and they should stay high until the population stunting reoccurs. It would be very interesting to see if this indeed is the case. The most likely avian host for the *Ligula* parasite in the lake is the Great Crested Grebe, and this species is only attracted to the lake after the roach population reaches a stunted population structure [16]. This is probably because the high juvenile density provides better fishing conditions for the birds. This effect could act to reinforce the occurrence of repeated epidemics.

Notwithstanding all these additional complexities, it is telling that already our basic model can exhibit the recurrent epidemics and the shifts between the stunted and non-stunted host population observed in the lake Slapton Ley data. Furthermore, we demonstrate that the introduction of a disease could act to decrease population stuntedness, which could potentially be of importance for fishery management [24].

A final topic we would like to address is the evolution of the parasite characteristics, in particular how it affects the fish host life history parameters [25]. Here, selection pressure potentially acts differently on different levels. Keeping the individual infected host in good condition seems advantageous, as parasite induced morbidity and mortality will in general not contribute to parasite fitness. However, if the parasite can increase the chance that the fish is eaten by the final bird host species, this would actually increase parasite fitness. We add the aspect of the parasite being able to keep the fish host population in the preferred stunted population state. How these different levels act in concert to shape the life history of the parasite and the host will be the scope of further investigations.

## References

[1] Grenfell BT, Dobson AP, editors. Ecology of Infectious Diseases in Natural Populations. Cambridge, UK: Cambridge University Press, Cambridge; 1995.

[2] Thomas F, François Renaud F, Guégan JF, editors. Parasitism and Ecosystems. Oxford, UK: Oxford University Press; 2005.

[3] Osfeld RS, Keesing F, Eviner VT, editors. Infectious Disease Ecology: Effects of Ecosystems on Disease and of Disease on Ecosystems. Princeton, NJ: Princeton University Press; 2008.

[4] Ohlberger J, Langangen Ø, Edeline E, Claessen D, Winfield IJ, Stenseth NC, et al Stage-specific biomass overcompensation by juveniles in response to increased adult mortality in a wild fish population. Ecology 2001; 92: 2175–2182. 10.1890/11-0410.1 22352155

[5] Collinge SK, Ray C, editors. Disease Ecology: Community structure and pathogen dynamics. Oxford, UK: Oxford University Press; 2006.

[6] Amundsen PA, Lafferty KD, Knudsen R, Primicerio R, Klemetsen A, Kuris AM. Food web topology and parasites in the pelagic zone of a subarctic lake. J Anim Ecol. 2009; 78: 563–72. 10.1111/j.1365-2656.2008.01518.x 19175443

[7] Kennedy CR. The ecology of parasites of freshwater fishes: the search for patterns. Parasitology 2009; 136: 1653–1662. 10.1017/S0031182009005794 19366479

[8] Keesing F, Belden LK, Daszak P, Dobson A, Harvell CD, Holt RD, et al Impacts of biodiversity on the emergence and transmission of infectious diseases. Nature 2010; 468: 647–652. 10.1038/nature09575 21124449 PMC7094913

[9] Heins DC, Baker JA, Green DM. Processes Influencing the Duration and Decline of Epizootics in Schistocephalus solidus. J Parasitol. 2011; 97: 371–376. 10.1645/GE-2699.1 21506858

[10] Loot G, Franscisco P, Santoul F, Lek S, Guégan JF. The three hosts of the Ligula intestinalis (Cestoda) life cycle in Lavernose-Lacasse gravel pit, France. Archiv für Hydrobiologie 2001; 152: 511–525.

[11] Kennedy CR, Shears PC, Shears JA. Long-term dynamics of Ligula intestinalis and roach Rutilus rutilus: a study of three epizootic cycles over thirty-one years. Parasitology 2001; 123: 257–69. 11578089 10.1017/s0031182001008538

[12] Carter V, Pierce R, Dufour S, Arme C, Hoole D. The tapeworm Ligula intestinalis (Cestoda: Pseudophyllidea) inhibits LH expression and puberty in its teleost host, Rutilus rutilus. Reproduction 2005; 130: 939–45. 16322554 10.1530/rep.1.00742

[13] Loot G, Lek S, Brown SP, Guégan JF. Phenotypic modification of roach (Rutilus rutilus L.) infected with Ligula intestinalis (Cestoda: Pseudophyllidea). J Parasitol. 2001; 87: 1002–1010. 11695356 10.1645/0022-3395(2001)087[1002:PMORRR]2.0.CO;2

[14] Hoole D, Carter V, Dufour S. Ligula intestinalis (Cestoda: Pseudophyllidea): an ideal fish-metazoan parasite model? Parasitology 2010; 137: 425–38. 10.1017/S0031182010000107 20163752

[15] Kennedy CR. The fish of Slapton Ley. Field Studies 1996; 8: 685–697.

[16] Dobson AP, Lafferty KD, Kuris AM. Parasites and Food Webs In: Dunne J, Pascual M, editors. Ecological Networks: Linking Structure to Dynamics. Oxford, UK: Oxford University Press; 2005 pp. 119–135

[17] de Roos AM, Persson L. Population and Community Ecology of Ontogenetic Development Monographs in Population Biology, Levin S, Horn HS, editors. Princeton, NJ: Princeton University Press; 2013.

[18] van Kooten T, de Roos AM, Persson L. Bistability and an Allee effect as emergent consequences of stage-specific predation. J Theor Biol. 2005; 237: 67–74. 15935390 10.1016/j.jtbi.2005.03.032

[19] Boerlijst MC, Oudman T, de Roos AM. Catastrophic Collapse can occur without Early Warning: Examples of Silent Catastrophes in Structured Ecological Models. PloS One 2013; 8: e62033 10.1371/journal.pone.0062033 23593506 PMC3623932

[20] Dhooge A, Govaerts W, Kuznetsov YA. MatCont: A MATLAB package for numerical bifurcation analysis of ODEs. ACM TOMS 2003; 29: 141–164

[21] Kuznetsov YA. Elements of Applied Bifurcation Theory. New York: Springer-Verlag; 2004.

[22] de Roos AM, Persson L. Size-dependent life-history traits promote catastrophic collapses of top predators. Proc Natl Acad Sci U S A 2002; 99: 12907–12912. 12237404 10.1073/pnas.192174199 PMC130558

[23] de Roos AM, Persson L, Thieme HR. Emergent Allee effects in top predators feeding on structured prey populations. Proc Roy Soc Lond B 2003; 270: 611–618. 10.1098/rspb.2002.2286 PMC1691284 12769461

[24] Ylikarjula J, Heino M, Dieckmann U. Ecology and adaptation of stunted growth in fish. Evol. Ecol. 1999; 13: 433–453

[25] Loot G, Park YS, Lek S, Brosse S. Encounter rate between local population shapes host selection in complex parasite life cycle. Biol J Linn Soc. 2006; 89: 99–106

